# Pediatric trauma patients in Swedish ambulance services -a retrospective observational study of assessments, interventions, and clinical outcomes

**DOI:** 10.1186/s13049-024-01222-0

**Published:** 2024-06-05

**Authors:** Glenn Larsson, Sanna Larsson, Viktoria Strand, Carl Magnusson, Magnus Andersson Hagiwara

**Affiliations:** 1 Centre for Prehospital Research, Faculty of Caring Science, Work Life and Social Welfare, University of Borås, Borås, SE-501 90 Sweden; 2 Department of Prehospital Emergency Care, Sahlgrenska University Hospital, Gothenburg, Sweden; 3 PICTA, Prehospital Innovation arena, Lindholmen Science Park, Gothenburg, Sweden

**Keywords:** Pediatric trauma, Prehospital assessment, Interventions, Prehospital care, Emergency medical services, Clinical outcomes, Retrospective observational study, Pediatric injuries

## Abstract

**Background:**

Pediatric trauma patients constitute a significant portion of the trauma population treated by Swedish Emergency Medical Services (EMS), and trauma remains a notable cause of death among Swedish children. Previous research has identified potential challenges in prehospital assessments and interventions for pediatric patients. In Sweden, there is limited information available regarding pediatric trauma patients in the EMS. The aim of this study was to investigate the prevalence of pediatric trauma patients within the Swedish EMS and describe the prehospital assessments, interventions, and clinical outcomes.

**Methods:**

This retrospective observational study was conducted in a region of Southwestern Sweden. A random sample from ambulance and hospital records from the year 2019 was selected. Inclusion criteria were children aged 0–16 years who were involved in trauma and assessed by EMS clinicians.

**Results:**

A total of 440 children were included in the study, representing 8.4% of the overall trauma cases. The median age was 9 years (IQR 3–12), and 60.5% were male. The leading causes of injury were low (34.8%) and high energy falls (21%), followed by traffic accidents. The children were assessed as severely injured in 4.5% of cases. A quarter of the children remained at the scene after assessment. Complete vital signs were assessed in 29.3% of children, and 81.8% of children were assessed according to the ABCDE structure. The most common intervention performed by prehospital professionals was the administration of medication. The mortality rate was 0.2%.

**Conclusions:**

Pediatric trauma cases accounted for 8.4% of the overall trauma population with a variations in injury mechanisms and types. Vital sign assessments were incomplete for a significant proportion of children. The adherence to the ABCDE structure, however, was higher. The children remained at the scene after assessment requires further investigation for patient safety.

**Supplementary Information:**

The online version contains supplementary material available at 10.1186/s13049-024-01222-0.

## Background

Trauma is a leading cause of death and disability among children worldwide [1]. Road traffic accidents and falls are common mechanisms of injury among pediatric patients globally [2]. In Sweden, severely injured pediatric patients account for approximately 14% of all severe trauma cases [3]. Fortunately, Sweden boasts relatively low mortality rates compared to other countries [4]. However, trauma, especially that associated with road traffic accidents, remains a significant cause of death [3].

Emergency Medical Services (EMS) play a crucial role in providing initial care, including assessments, stabilization, triage, and transportation to definitive care for injured children [5]. EMS clinicians encounter injured children less frequently than adults, and the perceived special social value of children contributes to the notion that the care of children is fundamentally different from that of adults, leading to stress and a sense of high stakes [6]. Research has identified potential challenges and shortcomings in assessments and interventions for pediatric patients. A study by Bankole et al. [7] revealed a higher complication rate in endotracheal intubation and intravenous access among children with severe head injuries compared to adults. Additionally, a study by Ramgopal et al. [8] focused on the disparity in assessment practices between adults and children, revealing significantly lower rates of vital signs assessments in pediatric patients compared to adult patients. Additional challenges that contribute to clinical difficulties in assessing pediatric patients include communication barriers, specialized dosages, and the need for smaller equipment [6]. Consequently, these factors collectively increase the risk of adverse events and patient harm in pediatric care [9].

Despite a growing body of knowledge in the trauma field, research on prehospital care for pediatric injuries is limited [10, 11]. Existing studies often focus on severely injured children, multi-trauma cases, and traumatic brain injuries [12]. However, a significant number of pediatric trauma patients need medical attention even if they are not classified as severely injured [13]. The current knowledge in Sweden primarily relies on data collected by the national trauma register, which solely includes severely injured children and lacks comprehensive information on prehospital interventions [3]. As a result, there is a significant gap in our understanding of the full extent of pediatric trauma patients who interact with the EMS in Sweden, regarding the prevalence of pediatric trauma, prehospital assessments, interventions, and clinical outcomes. Expanding knowledge in this area can help identify areas of improvement, enhance the professional growth of EMS clinicians, and improve prehospital care for pediatric trauma patients. Therefore, the aim of this study was to investigate the prevalence of pediatric trauma patients within the Swedish EMS and describe the prehospital assessments, interventions, and clinical outcomes.

## Materials and methods

### Design of the study

This study was a one-year retrospective observational study of pediatric trauma patients, in which EMS and hospital medical records from the year 2019 were reviewed. The design and methodology of the study were based on the model for methods in chart review studies by Kaji et al. [14], and the study was guided by the STROBE protocol [15].

### Settings and population

The study was conducted in a region of Southwestern Sweden covering an area of 23,942 km² and inhabited by approximately 1.7 million people, with 21% of them being children aged 0–16 years old. The region is divided into five hospital administrations and 10 hospitals with emergency departments. All of these hospitals have pediatric departments. Only one hospital is a specialized pediatric hospital with major pediatric trauma capacity and pediatric intensive care. The specialist hospital is located in the western part of the region, which may entail long transport distances to specialized pediatric care. There are 46 ambulance stations equipped with approximately 110 ambulance units in the region. In the year 2019, the EMS in the region carried out 173,536 ambulance assignments. Among these, approximately 10,065 (8.5%) involved children aged 0–16 years [16, 17]. In Southwestern Sweden, as well as in the rest of the country, the EMS is staffed by registered nurses who have often completed postgraduate diplomas in prehospital emergency care. The typical ambulance crew consists of either two registered nurses or one registered nurse and one emergency medical technician (EMT) [18]. In the region, there is one physician-staffed car and one physician-staffed helicopter. None of the patients in the study were assessed by these units.

The EMS in the region conducts prehospital care guided by regional prehospital guidelines. These guidelines empower EMS clinicians to independently administer around 30 medications.

Since there is no uniform description in Sweden of what should be included in a specialist education in ambulance care, nor which internal trainings should be included for ambulance nurses, we can assume that the competence regarding pediatric patients varies. A few ambulance organizations in Sweden provide pediatric training such as Pediatric Education for Prehospital Professionals (PEPP) [19]. Most provide training in pediatric cardiopulmonary resuscitation.

The Emergency Medical Dispatch Centre (EMDC) plays a crucial role in efficiently deploying EMS resources. To prioritize patient care, the EMDC utilizes a Dispatch Medical Index (DMI), which considers the severity of the patient’s condition. Ambulances are dispatched based on three priorities: priority 1 (for acute life-threatening situations requiring immediate attention, dispatched with blue lights and sirens), priority 2 (acute but not life-threatening situations requiring prompt medical attention), and priority 3 (other missions in need of ambulance transport but can wait) [20].

Throughout the study, all EMS organizations used the pediatric Rapid Emergency Triage and Treatment System (RETTS-P), consisting of two parts: Vital Signs (VS) and Emergency Signs and Symptoms (ESS). Severity is assessed based on the highest priority color of VS or ESS, resulting in a priority assessment of the patient. The triage colors in RETTS-P are as follows: red indicates a life-threatening condition, orange indicates a potentially life-threatening condition requiring urgent attention, and yellow and green indicate that the patient is not facing any immediate medical risk and can wait to see an emergency physician [21]. RETTS-P has been validated in a previous study [22]. In the study, it was found that RETTS-P red and orange had a sensitivity of 66.7% for detecting severely ill patients and a specificity of 67%. This corresponds to an under triage of 33.3% and an over triage of 33%.

## Materials

### Data sampling

**Fig. 1:**
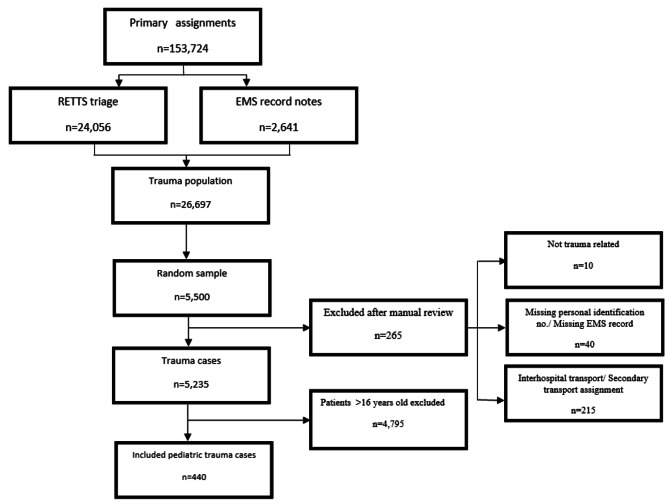
Inclusion flow chart

In 2019, a total of 153,724 primary EMS assignments were conducted between January 1st and December 31st. To be included in the study, patients had to meet the following criteria: being 0–16 years of age, having been involved in trauma, and having been assessed as requiring prehospital care. The decision to include children up to 16 years of age was influenced by the modified Spinal Motion Restriction (SMR) guidelines in Sweden, which consider the physiological differences in this age group [23]. Additionally, in the Southwestern region of Sweden, specific pediatric guidelines are followed for the treatment of children up to 16 years of age. Of the total primary missions, 24,056 were related to trauma according to the ESS code. An additional (*n* = 2,641) assignment where a RETTS code had not been registered was included in the study to avoid overlooking any potential time-critical patients who may not have had time for the triage system to be used in the prehospital setting due to the patient’s critical condition. In total, 26,697 patients were identified as having trauma-related injuries, making up 17.4% of all primary assignments conducted that year. In the second phase, a random sample of 5,500 EMS records was drawn from the total number of identified trauma assignments. The sample was drawn based on assignments and the proportional distribution of the five EMS organizations in the region, with 265 assignments excluded. Out of the remaining 5,235 patients, 440 were children aged 0–16 years and were included in the study (Fig. 1). The medical records underwent thorough manual reviews conducted by designated nurses following the guidelines outlined by Kaji et al. [14].

The following data were retrieved from prehospital records: ambulance assignment number, patient personal identification number, date and time, gender, age, dispatch center priority, place of injury, type of injury, mechanism of injury, vital signs, pain, RETTS, triage color, transport destination, assessment according to ABCDE-structure, and treatment details, including interventions for free airway and bleeding, administration of analgesics (paracetamol [acetaminophen], non-steroidal anti-inflammatory drugs [NSAIDs], opiates, and ketamine), other medications, spinal motion restriction (SMR), and fracture stabilization. The hospital’s electronic record system provided data on past medical history, hospital admission, treatment received, length of hospital stays, and discharge destination and mortality information.

### Statistical analysis

Outcome data were summarized using descriptive statistics and presented with numbers, percentages, medians, and interquartile ranges. Patients were categorized into four age groups: infants (0–12 months), younger children (1–5 years), older children (6–11 years), and adolescents (12–16 years). Fisher’s exact tests were employed to compare these age groups. All tests were two-sided, and a p-value of < 0.05 was considered statistically significant. The data processing and statistical analysis were carried out using IBM SPSS version 28.0.0.

### Ethics

This study received approval from the Swedish Ethical Review Authority in Stockholm, Sweden (Dnr 2020 − 00490) and was conducted in accordance with ethical guidelines and regulations. The head of operations of the participating organizations granted approval for the study before reviewing the medical records. Informed consent is generally not required for observational studies of this nature. The current study adheres to the ethical principles outlined in the Helsinki Declaration [24].

## Results

Out of the 5,235 EMS assignments registered as trauma, 440 patients (8.4%) were 16 years old or younger. The median age was 9 years (IQR 3–12), and 266 (60.5%) were male. It should be noted that gender data are missing for five children. Regarding EMDC dispatch, 191 (43.5%) were assigned priority 1. The most common mechanisms of injury were low-energy falls (*n* = 150; 34.1%), followed by high-energy falls (*n* = 90; 20.5%). Among road traffic accidents, the largest proportion was caused by bicycle accidents (*n* = 25; 5.7%). Out of the total cases, 181 (41.1%) did not have any documented injuries, while 31 (7%) children had multiple injuries. The most frequent injuries were lacerations or wounds (*n* = 102; 23.2%), followed by hematoma or swelling (*n* = 78; 17.7%), and closed fractures (*n* = 66; 15%). Dislocation injuries and closed fractures were more common among older children.

(*p* < 0.001), while burns were more frequent among younger children (*p* = 0.003). The most common place of injury was the patient’s home (*n* = 190; 43.2%), particularly among younger children aged 1–5 years (*n* = 101; 71.6%) (Table 1).

**Table 1:** Distribution of age, EMDC priority, mechanism of injury, type of injury, and place of injury

Epidemiology	All patients	0–12 mon	1–5 years	6–11 years	12–16 years	*P*-value
**Total n (%)**	440 (100)	25 (5.7)	141 (32)	136 (30.9)	138 (31.4)	
**Dispatcher priority n (%)**						
Priority 1	191(43.4)	19 (76)	64 (45.4)	52 (38.2)	56 (40.6)	0.01
Priority 2	244 (55.5)	6 (24)	75 (53.2)	82 (60.3)	81(58.7)	0.01
Priority 3	4 (0.9)	0 (0)	2 (1.4)	1 (0.7)	1(0.7)	0.88
Missing	1(0.2)			1(0.7)		
**Mechanism of injury n (%)**						
Road traffic accident	62 (14)	2 (8)	11(7.8)	19 (14)	30 (21.7)	< 0.01
Stabbed by sharp object	6 (1.4)	0 (0)	3 (2.1)	3 (2.2)	0 (0)	0.31
Hit by blunt object	43 (9.8)	1 (4)	6 (4.3)	14 (10.3)	22 (15.9)	0.01
Low energy fall	150 (34.1)	6 (24)	59 (41.8)	46 (33.8)	39 (28.2)	0.07
High energy fall	90 (20.5)	4 (16)	29 (20.6)	36 (26.4)	21(15.2)	0.14
Other injury	75 (17)	8 (32)	29 (20.6)	15 (11)	23 (7, 16)	0.01
Missing	14 (2, 3)	4 (16)	4 (2.8)	3 (2)	3 (2)	
**Type of injury n (%)**						
Laceration, ulcer, wounds	102 (23.2)	7 (28)	37 (26.2)	34 (25)	24 (17.4)	0.19
Closed fracture	66 (15)	1 (4)	10 (7.1)	25 (18.4)	30 (21.7)	< 0.01
Open fracture	4 (0.9)	0 (0)	0 (0)	3 (2.2)	1(0.7)	0.26
Dislocation	14 (3.2)	0 (0)	0 (0)	2 (1.5)	12 (8.7)	< 0.01
Burn	10 (2.3)	1 (4)	8 (5.7)	1 (7.4)	0 (0)	< 0.01
Hematoma, swollen, abrasion	78 (17.7)	5 (22)	33 (23.4)	22 (16.2)	18 (13)	0.10
No documented injury	181 (41.1)	11 (44)	64 (45.4)	54 (39.7)	52 (37.7)	0.43
Missing	8 (1.8)		6 (4.3)		2 (1.4)	
**Place of injury n (%)**						
Home	190 (43.2)	12 (52.2)	101 (71.6)	56 (41.2)	21 (15.2)	< 0.01
Public place	97 (22)	10 (43.4)	18 (12.7)	31(22.7)	38 (27.5)	< 0.01
Sports arena	92 (20.9)	0 (0)	8 (5.7)	24 (17.6)	60 (43.4)	< 0.01
School	29 (6.6)	0 (0)	4 (2.8)	18 (13.2)	7(5.1)	< 0.01
Other	19 (4.3)	1 (4)	2 (1.4)	5 (3.7)	11 (8)	0.07
Missing	13 (3)	2 (8)	8 (5.6)	2 (1.5)	1 (0.7)	

### Prehospital assessment

The children were assessed according to RETTS-P, and 4.5% (*n* = 20) were prioritized as having life-threatening conditions, while 21% (*n* = 92) were deemed to have potentially life-threatening conditions. Complete vital signs were assessed in 129 (29.3%) of the children, and the assessment rate was significantly lower for children aged 1–5 years in four out of five vital signs: blood pressure, pulse rate, respiratory rate, and oxygen saturation (*p* < 0.001). Blood pressure had the lowest assessment rate, being assessed in 156 (35.5%) of all children. In 23 cases, vital signs were not assessed. In 360 (81.8%) cases, the children were assessed according to the ABCDE-structure. However, adherence to the ABCDE-structure was significantly lower (*p* < 0.001) in the 1–5 years age group. The ABCDE-structure tended to be performed slightly more often in critically injured children, conducted in 95% (*n* = 19) of the children assessed as having a life-threatening condition compared to 83% (*n* = 125) of the children who were assessed as not facing any immediate medical risk. Pain assessment was performed in 396 (90%) of the children, and 263 (66%) experienced pain (Table 2).

**Table 2:** Prehospital assessment - distribution of age, vital signs, ABCDE-structure, pain assessment, and RETTS priority after assessment

Assessments	All patients	0–12 mons	1–5 years	6–11 years	12–16 years	*P*-value
**Total n (%)**	440 (100)	25 (5.7)	141 (32)	136 (30.9)	138 (31.4)	
**Registration of vital signs n (%)**						
Complete vital sign registered	129 (29.3)	6 (24)	4 (2.8)	33 (24.3)	86 (62.3)	< 0.01
Blood pressure	156 (35.5)	8 (32)	6 (4.3)	44 (32.4)	98 (71)	< 0.01
Pulse rate	323 (73.4)	15 (60)	79 (56)	108 (79.4)	121 (87.7)	< 0.01
Respiratory rate	281(63.9)	13 (52)	60 (42.6)	96 (70.6)	112 (81.2)	< 0.01
Oxygen saturation	329 (74.8)	16 (64)	84 (59.6)	107 (78.7)	122 (88.4)	< 0.01
Level of Consciousness	370 (84.1)	22 (88)	108 (76.6)	119 (87.5)	121 (87.7)	0.03
**Adherence to ABCDE-concept n (%)**	360 (81.8)	21 (84)	102 (72.3)	115 (84.6)	122 (88.4)	< 0.01
Missing	8 (2.2)	3 (12)	4 (2.8)	1 (0.7)		
**Pain - yes**	263 (66.4)	4 (26.7)	43 (37.1)	96 (73.3)	120 (90)	< 0.01
**Priority n (%)**						
Red	20 (4.5)	8 (32)	7 (5)	2 (1.5)	3 (2.2)	< 0.01
Orange	92 (20.9)	2 (8)	23 (16.3)	32 (23.5)	35 (25.4)	0.56
Yellow	90 (20.5)	2 (8)	15 (10.6)	31(22.8)	42 (30.4)	0.01
Green	151 (34.3)	3 (12)	53 (37.6)	51(37.5)	44 (31.9)	0.01
Missing	87 (19.8)	10 (40)	43 (10.8)	20 (14.7)	14 (10.1)	

### Prehospital interventions

**Fig. 2:**
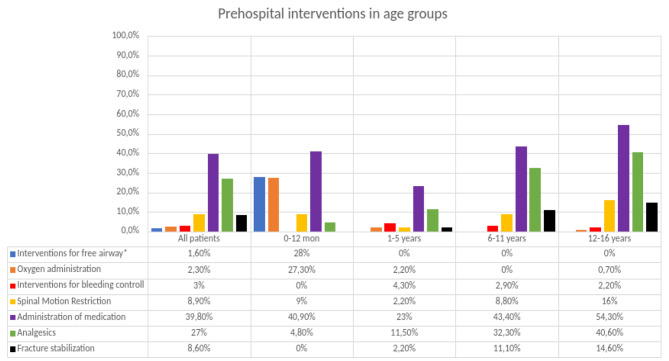
Prehospital intervention in age groups. Percentage of children within each age group who received a certain intervention

Interventions for a free airway were performed in seven (1.6%) children, all of whom were under one year of age. The most commonly performed interventions for a free airway were manually holding the airway open and clearing blood and secretions by suction. One child received a supraglottic airway, and 10 (2.3%) received oxygen. Bleeding control was performed in 13 children (3%), with all of them receiving a pressure dressing. Two children (0.5%) received fluid therapy. Spinal motion restriction (SMR) was performed in 39 (8.9%) children. The likelihood of performing SMR was significantly higher in older children and adolescents (*p* < 0.001). The most common intervention performed by prehospital professionals was the administration of medication. Analgesics were the most commonly administered medication, given to 117 (27%) of the children overall and to 111 (42.2%) of the children with documented pain. Fracture stabilization was performed in 38 (54.3%) of the children with suspected fractures and was more common among older children and adolescents (*p* < 0.001) (Fig. 2). Data on prehospital intervention were unavailable for seven children.

### Destination and definitive care

The majority of the injured children (*n* = 296; 67%) were transported to emergency departments. Almost one-quarter of the children (*n* = 108; 25%) remained at the scene. Of these had 10 (11%) complete vital signs documented and 88 (81.5%) were assessed according to the ABCDE structure. Bleeding control and analgesics administration was performed in three (0.2%) respectively 10 (11%) of the children. Low-energy falls was the most common mechanism of injury (*n* = 44; 40%) and 43% of these had no documented injuries. The most frequent injuries were wounds. Children aged 1–5 years were more likely to remain at the scene than other age groups (*p* < 0.001). Four children (0.9%) needed secondary ambulance transport within 72 h, and one was transported by own mode of transport to ED. Of these was two admitted to a hospital ward.

**Fig. 3:**
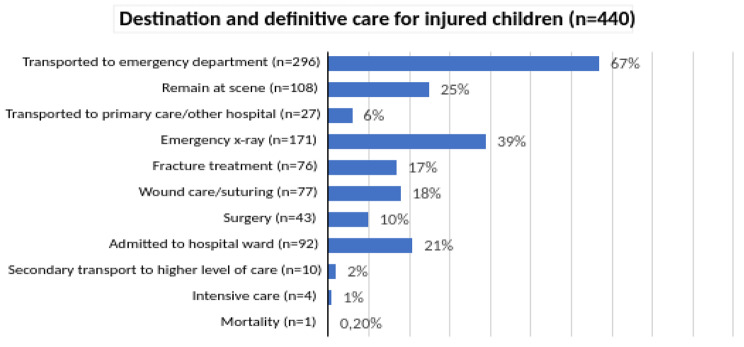
Destination and intervention at definitive care for injured children. For nine of the children, information about their destination was missing

In 92 (21%) cases, the children were admitted to a hospital ward, and four children (1%) required intensive care. Fractures were the most common reason for surgery (*n* = 36; 83.7%). One child died, resulting in a mortality rate of 0.2%. The child was lifeless upon EMS arrival at the scene and was pronounced dead at the hospital (Fig. 3).

## Discussion

This observational study revealed that pediatric trauma cases make up 8.4% of all trauma cases within the Swedish EMS. The majority of injured pediatric patients present with moderate and mild injuries, and an important finding was that a quarter of the children remained at the scene after assessment. Only 29.3% of the children had vital signs completely assessed, and although assessment according to the ABCDE-structure was conducted in 81.8% of the children, there was a significantly lower rate of both vital sign assessment and adherence to the ABCDE-structure in the age group of 1–5 years.

Moreover, despite assigning a high EMDC priority to the majority of cases, a substantial number of children were assessed by EMS clinicians as moderately injured or unharmed. This discrepancy aligns with previous studies that have shown inconsistencies between EMDC priority and EMS prioritization, leading to the overuse of ambulances [20]. In a systematic review focusing on the accuracy of EMDC in trauma cases, over-triage emerged as a notable issue, as highlighted by Bohm and Kurland [25]. Furthermore, a study by Nesje et al. [13] demonstrated that even EMS clinicians tend to over-triage when assessing injured children. It is acknowledged that a certain degree of over-triage is necessary to prevent missing any seriously injured children [20, 25]. In the present study, it was found that a significant proportion, approximately a quarter, of the children assessed remained at the scene. This finding aligns with previous studies conducted on pediatric patients, which have reported similar rates of non-conveyance. For instance, a systematic review focusing on non-conveyance patients revealed a non-conveyance rate ranging from 13.2 to 27.7%, for pediatric patients [26]. However, it is important to note that the review included all types of conditions. Due to different studies and design we are unable to compare our finding from a patient safety perspective regarding non-conveyed pediatric patients in the EMS.

This study reinforces the existing data on pediatric trauma, particularly with regards to the distribution between genders, mechanisms of injury, and type of injury. Consistent with previous studies, our study indicates that boys are more prone to injuries and that road traffic accidents and falls are the primary mechanisms of injury [2, 13, 27]. The incidence of penetrating trauma was 1.4%, which is in line with previous research conducted in Scandinavia [13]. Laceration and fractures were common types of injuries, in this study, as well as in other international epidemiologic studies [2, 27]. A recent study [28] of over 12,000 injured children found that fractures accounted for 21% of the injuries among children of all ages, with a peak in school-age children and that 25.9% of these fractures required surgical treatment. Our study supports the notion that the incidence of fractures increases with age and that fractures were the leading cause for surgery after trauma.

The results of this study highlight a disparity in prehospital assessment of pediatric trauma patients among different age groups. Specifically, children aged 1–5 years had lower rates of vital sign assessment and assessment according to the ABCDE-structure compared to infants and older children. Recent studies have revealed suboptimal adherence to the ABCDE-structure among healthcare professionals in critically ill or injured children [29, 30]. The present study found a relatively high adherence to the ABCDE-structure overall but significant differences between age groups that have not been well-investigated before. It is reasonable to believe that the low adherence to the ABCDE-structure is linked to the low rate of complete vital sign assessment in these children. This is consistent with previous studies that have documented a low rate of complete vital sign assessment in small children, with a decreasing probability for complete assessment as the child’s age decreases [8, 31, 32]. The reasons for incomplete vital signs assessments in young pediatric patients appear to be multifaceted, as previous studies have emphasized various contributing factors. These include communication difficulties, anxiety, inadequate availability of proper equipment, insufficient knowledge, and experience in conducting pediatric assessments, and concerns about distressing the child [33,34, 35]. The present study found that blood pressure was the least assessed vital sign (36.1%), particularly in children aged 1–5 years, where only 4.3% of those children had recorded information. While it may be reasonable not to take blood pressure as the sole indicator of severity, other vital signs should be considered in the assessment of pediatric trauma patients and can help EMS clinicians make a more accurate decision [36].

Bradman and Maconochie [37] discovered that a pediatric triage tool based on vital signs exhibited a low sensitivity, limiting its effectiveness of predicting the need for emergency department admission. A Swedish study [38] found that the assessment of children by EMS nurses generally aligns with the clinical situation, indicating appropriate care levels, despite incomplete utilization of vital signs. However, previous studies showed that missing physiological data during trauma incidents has an association with poor outcome [39]. The reason for this could be that vital parameters are not documented in the most critical patient cases, as other tasks are prioritized.

Significant differences were observed in required interventions across different age groups. Specifically, small children required airway and breathing interventions more frequently, which could be due to their anatomic and physiologic divergence. Compared to adults and older children, the anatomy of the small children’s airways is distinct which increases the risk of airway obstruction. Moreover, young children have a significantly higher metabolic rate and therefore a higher oxygen demand [5, 40]. On the other hand, older children were more likely to require fracture treatment and spinal motion restriction, aligning with previous investigations that have consistently identified fractures and SMR as the most frequently observed injuries among school-aged children [28].

The most common intervention performed by EMS clinicians was medication administration, particularly analgesics. However, less than half of the children experiencing pain (42.6%) received analgesics. This finding is supported by a systematic review conducted by Samuel et al. [41] which explored prehospital pain treatment in children and revealed a low rate of analgesic administration, ranging from a few percent up to 15% among children with pain. However, a more recent study [42] reported a higher analgesics administration rate of 32%, among children in the prehospital setting. Although this rate is higher than the previous studies, it is still lower than the rate observed in the present study, indicating that there is room for improvement in providing adequate pain management for pediatric patients in the prehospital setting. It is worth noting that all ambulances in Sweden are staffed with at least one registered nurse and often one nurse with additional education in prehospital care. Therefore, it is plausible that additional education makes EMS clinicians more confident in administering medications, which could explain the higher rate of analgesics given in our study.

### Strengths and Limitations

A strength of this study is the utilization of a random sample, which helps ensure an equal chance of inclusion for each member of the target population. However, there are some limitations that warrant acknowledgment. Firstly, the study was conducted in a single geographic location, potentially limiting generalizability to other regions with different environmental factors and work cultures. Another limitation is the reliance on self-reported data, which may be subject to recall bias. An additional limitation of the study arises from the use of the RETTS system rather than other well-established systems, such as the Injury Severity Score (ISS), when attempting to compare the distribution of child injury severity across different countries. Additionally, the review of medical records may not always provide a complete and accurate reflection of the events that occurred at the scene of the accident. Moreover, although the records were reviewed by experienced nurses, there is a risk of errors and different interpretations. Nonetheless, this approach enabled a more comprehensive evaluation of medical records and allowed for the analysis of large amounts of textual data, moving beyond checkboxes or simple data points. As the medical record review was descriptive in nature, the study cannot comment on patient safety risks or adverse events. For that, medical record review with trigger tools or similar instruments is required.

## Conclusion

In the present study, children accounted for 8.4% of the trauma population. Injured children emerged as a diverse group demonstrating variations in the mechanism and type of injuries observed, as well as differences in the assessments and interventions conducted by EMS clinicians across different age groups. Notably, only 29.3% of the children had their vital signs completely assessed, while 81.8% of them were assessed according to the ABCDE-structure. However, children aged 1–5 years had significantly lower rates of both vital sign assessment and assessment according to the ABCDE-structure. Additionally, it is noteworthy that a quarter of the children remained at the scene following their assessment. This particular group represents an unexplored aspect that demands further investigation, particularly from a patient safety perspective. Based on the current findings, there are several opportunities for enhancing prehospital care for pediatric trauma patients. These include the implementation of evidence-based guidelines, ongoing research initiatives, targeted educational programs, and regular practice and simulations for EMS clinicians.

### Electronic supplementary material

Below is the link to the electronic supplementary material.

Supplementary Material 1

## Data Availability

The datasets generated and/or analyzed during the current study are not publicly available due to the General Data Protection Regulation (GDPR) unclear rules regarding research data but are available from the corresponding author on reasonable request.

## Abbreviations

EMS: Emergency Medical Services
EMT: Emergency Medical Technician
EMDC: Emergency Medical Dispatch Centre
DMI: Dispatch Medical Index
RETTS-P: Pediatric Rapid Emergency Triage and Treatment System
VS: Vital Signs
ESS: Emergency Signs and Symptoms
SMR: Spinal Motion Restriction
ISS: Injury Severity Score

## References

[1] Centers for Disease Control and Prevention (CDC). Leading causes of death and data visualization. 2020. https://wisqars.cdc.gov/data/lcd/home. Accessed 2 mars 2023.

[2] Bradshaw CJ, Bandi AS, Muktar Z, Hasan MA, Chowdhury TK, Banu T (2018). International Study of the epidemiology of paediatric trauma: PAPSA Research Study. World J Surg.

[3] Swedish Trauma R, Årsrapport. 2021. 2022 https://rcsyd.se/swetrau/wp-content/uploads/sites/10/2022/06/A%CC%8Arsrapport-SweTrau-2021.pdf. Accessed 2 mars 2023.

[4] Bäckström D, Steinvall I, Sjöberg F (2017). Change in child mortality patterns after injuries in Sweden: a nationwide 14-year study. Eur J Trauma Emerg Surg.

[5] Seid T, Ramaiah R, Grabinsky A (2012). Pre-hospital care of pediatric patients with trauma. Int J Crit Illn Inj Sci.

[6] Jeruzal JN, Boland LL, Frazer MS, Kamrud JW, Myers RN, Lick CJ (2019). Emergency Medical Services Provider perspectives on Pediatric calls: a qualitative study. Prehosp Emerg Care.

[7] Bankole S, Asuncion A, Ross S, Aghai Z, Nollah L, Echols H (2011). First responder performance in pediatric trauma: a comparison with an adult cohort. Pediatr Crit Care Med.

[8] Ramgopal S, Elmer J, Escajeda J, Martin-Gill C (2018). Differences in Prehospital Patient Assessments for Pediatric Versus Adult patients. J Pediatr.

[9] Meckler G, Leonard J, Hoyle J (2014). Pediatric Patient Safety in Emergency Medical services. Clin Pediatr Emerg Med.

[10] Groner JI, Phuong J, Price MA, Bixby PJ, Ehrlich PF, Burd RS (2022). Developing a National Trauma Research Action Plan: results from the Pediatric Research Gap Delphi Survey. J Trauma Acute Care Surg.

[11] Browne LR, Shah MI, Studnek JR, Farrell BM, Mattrisch LM, Reynolds S (2016). 2015 Pediatric Research priorities in Prehospital Care. Prehosp Emerg Care.

[12] Jeppesen E, Iversen VV, Hansen IS, Reierth E, Wisborg T (2020). Trauma research in the nordic countries, 1995–2018 - a systematic review. Scand J Trauma Resusc Emerg Med.

[13] Nesje E, Valøy NN, Krüger AJ, Uleberg O (2019). Epidemiology of paediatric trauma in Norway: a single-trauma centre observational study. Int J Emerg Med.

[14] Kaji AH, Schriger D, Green S (2014). Looking through the Retrospectoscope: reducing Bias in Emergency Medicine Chart Review studies. Ann Emerg Med.

[15] Cheng A, Kessler D, Mackinnon R, Chang TP, Nadkarni VM, Hunt EA (2016). Reporting guidelines for health care simulation research: extensions to the CONSORT and STROBE statements. Adv Simul.

[16] Västra Götalandsregionen. Demografi i Västra Götalandsregionen. 2021. https://www.vgregion.se/halsa-och-vard/. Accessed 2 mars 2023.

[17] Nysam, Ambulanssjukvård- Nyckeltal. 2019. 2020. https://nysam.com/images/Rapporter/Ambulanssjukvard_2019_Nysamrapport.pdf. Accessed 2 mars 2023.

[18] Lindström V, Bohm K, Kurland L (2015). Prehospital care in Sweden. Notf Rett Med.

[19] Fuchs S, McEvoy M (2020). Pediatric education for prehospital professionals.

[20] Khorram-Manesh A, Lennquist Montán K, Hedelin A, Kihlgren M, Örtenwall P (2011). Prehospital triage, discrepancy in priority-setting between emergency medical dispatch centre and ambulance crews. Eur J Trauma Emerg Surg.

[21] Ødegård SS, Tran T, Næss-Pleym LE, Risnes K, Døllner H (2021). A validity study of the rapid emergency triage and treatment system for children. Scand J Trauma Resusc Emerg Med.

[22] Magnusson C, Herlitz J, Karlsson T, Jiménez-Herrera M, Axelsson C (2019). The performance of the EMS triage (RETTS-p) and the agreement between the field assessment and final hospital diagnosis: a prospective observational study among children < 16 years. BMC Pediatr.

[23] Regionernas Ömsesidiga Försäkringsbolag (LÖF). Spinal rörelsebegränsning vid trauma. 2022. https://lof.se/filer/Spinal-rorelsebegransning-vid-trauma.pdf. Accessed 2 mars 2023.

[24] World Medical Association Declaration (2013). Of Helsinki: ethical principles for medical research involving human subjects. JAMA.

[25] Bohm K, Kurland L (2018). The accuracy of medical dispatch - a systematic review. Scand J Trauma Resusc Emerg Med.

[26] Ebben RHA, Vloet LCM, Speijers RF, Tönjes NW, Loef J, Pelgrim T (2017). A patient-safety and professional perspective on non-conveyance in ambulance care: a systematic review. Scand J Trauma Resusc Emerg Med.

[27] Aoki M, Abe T, Saitoh D, Oshima K, Epidemiology (2019). Patterns of treatment, and mortality of Pediatric Trauma patients in Japan. Sci Rep.

[28] Cintean R, Eickhoff A, Zieger J, Gebhard F, Schütze K (2023). Epidemiology, patterns, and mechanisms of pediatric trauma: a review of 12,508 patients. Eur J Trauma Emerg Surg.

[29] Olgers TJ, Dijkstra RS, Drost-de Klerck AM, Ter Maaten JC (2017). The ABCDE primary assessment in the emergency department in medically ill patients: an observational pilot study. Neth J Med.

[30] Linders M, Binkhorst M, Draaisma JMT, van Heijst AFJ, Hogeveen M (2021). Adherence to the ABCDE approach in relation to the method of instruction: a randomized controlled simulation study. BMC Emerg Med.

[31] Nielsen VML, Søvsø MB, Kløjgård TA, Skals RG, Corfield AR, Bender L (2023). Prehospital vital sign monitoring in paediatric patients: an interregional study of educational interventions. Scand J Trauma Resusc Emerg Med.

[32] Shinohara M, Muguruma T, Toida C, Gakumazawa M, Abe T, Takeuchi I (2022). The association between age and vital signs documentation of trauma patients in prehospital settings: analysis of a nationwide database in Japan. BMC Emerg Med.

[33] Hewes H, Hunsaker S, Christensen M, Whitney J, Dalrymple T, Taillac P (2016). Documentation of pediatric vital signs by EMS providers over time. J Pediatr Surg.

[34] Owusu-Ansah S, Moore B, Shah MI, Gross T, Brown K, Gausche-Hill M, et al. Pediatric Readiness in Emergency Medical services systems. Pediatrics. 2020;145. 10.1542/peds.2019-3308. 10.1542/peds.2019-3308 31857378

[35] Guise J-M, Hansen M, O’Brien K, Dickinson C, Meckler G, Engle P, et al. Emergency medical services responders’ perceptions of the effect of stress and anxiety on patient safety in the out-of-hospital emergency care of children: a qualitative study. BMJ Open. 2017;7. 10.1136/bmjopen-2016-014057. 10.1136/bmjopen-2016-014057 PMC5337745 28246139

[36] Fernandez A, Benito J, Mintegi S (2017). Is this child sick? Usefulness of the Pediatric Assessment Triangle in emergency settings. J Pediatr.

[37] Bradman K, Maconochie I (2008). Can paediatric early warning score be used as a triage tool in paediatric accident and emergency?. Eur J Emerg Med.

[38] Johansson H, Lundgren K, Hagiwara MA (2022). Reasons for bias in ambulance clinicians’ assessments of non-conveyed patients: a mixed-methods study. BMC Emerg Med.

[39] Laudermilch DJ, Schiff MA, Nathens AB, Rosengart MR (2010). Lack of Emergency Medical services documentation is Associated with poor patient outcomes: a validation of audit filters for Prehospital Trauma Care. J Am Coll Surg.

[40] Overly FL, Wills H, Valente JH (2014). Not just little adults’ - a pediatric trauma primer. R I Med J.

[41] Samuel N, Steiner IP, Shavit I (2015). Prehospital pain management of injured children: a systematic review of current evidence. Am J Emerg Med.

[42] Pilbery R, Miles J, Bell F (2019). A service evaluation of paediatric pain management in an English ambulance service. Br Paramed J.

