# Supplementary material for: Pediatric trauma patients in Swedish ambulance services -a retrospective observational study of assessments, interventions, and clinical outcomes
Source: Scand J Trauma Resusc Emerg Med. 2024 Jun 5;32:51. doi: 10.1186/s13049-024-01222-0 (PMC11151517; doi:10.1186/s13049-024-01222-0)
Supplement: Supplementary file 1 — Supplementary Material 1 [file 13049_2024_1222_MOESM1_ESM.docx]

| **Reviewer comments** | **Author answer** |
| --- | --- |
| Reviewer #1: |  |
| Thank you for your alterations following the review and for the clear responses. I appreciate the additional narrative around some of the elements as requested, which gives your article greater appeal outside Sweden. | Best reviewer, thank you for your valuable comments and assistance with the manuscript. |
| Reviewer #2: |  |
| P2L59 You write "An random sample". Please write "A random sample" | Thank you, it has now been changed. |
| P3L73-L74: The sentence is unclear to me. How can a prevalence indicate "variations in injury mechanisms and types" | This is a linguistic mistake. The sentence has now been changed to “Pediatric trauma cases accounted for 8.4% of the overall trauma population, with a indicating variations in injury mechanisms and types.” |
| P3L75: You present a result in the Conclusion not mentioned under Results, i.e. "particularly in the 1-5-year age group". Please, either delete the sentence or move it to Results. | The sentence has been removed, and the text adjusted to align with the results. |
| P6L168: The sentence: "Of these assignments (n=24,056), related to trauma, which consisted of a RETTS code" does not make sense. Please correct | We agree, and the sentence is changed to “Of the total primary missions, 24,056 were related to trauma according to the ESS code.” |
| P7L195: p<0.005. Why do you choose p<0.005 in stead of p<0.05? Bonferroni correction? | Thank you for noticing this. Typo corrected to 0.05. |
| P12L320: As a reader I still get the impression that "....the presence of incomplete prehospital assessments can poses a patient safety risk" (you own words). As mentioned in my first review, you have to rephrase or delete the sentence, so the reader is not left with an impression of a causal relationship between missing physiological measurements and outcome. This is simple not a correct interpretation of an association. It is probably the other way around (but we do not know), that in dying patients, eg. traumatic cardiac arrest, the measurement of vital signs are not prioritized by the treating clinicians. | We agree. We have removed the sentence about increased patient safety risk and added “The reason for this could be that vital parameters are not documented in the most critical patient cases, as other tasks are prioritized.”  Thank you for the work you've put into our manuscript, which has been improved. |
